# Association of Elective and Emergency Cesarean Delivery With Early Childhood Overweight at 12 Months of Age

**DOI:** 10.1001/jamanetworkopen.2018.5025

**Published:** 2018-11-21

**Authors:** Meijin Cai, See Ling Loy, Kok Hian Tan, Keith M. Godfrey, Peter D. Gluckman, Yap-Seng Chong, Lynette Pei-Chi Shek, Yin Bun Cheung, Ngee Lek, Yung Seng Lee, Shiao-Yng Chan, Jerry Kok Yen Chan, Fabian Yap, Seng Bin Ang

**Affiliations:** 1 Duke-NUS Medical School, Singapore, Singapore; 2 Department of Reproductive Medicine, KK Women’s and Children’s Hospital, Singapore, Singapore; 3 Department of Maternal Fetal Medicine, KK Women’s and Children’s Hospital, Singapore, Singapore; 4 Medical Research Council Lifecourse Epidemiology Unit, University of Southampton, Southampton, United Kingdom; 5 National Institute for Health Research Southampton Biomedical Research Centre, University of Southampton and University Hospital Southampton National Health Service Foundation Trust, Southampton, United Kingdom; 6 Liggins Institute, University of Auckland, Auckland, New Zealand; 7 Brenner Centre for Molecular Medicine, Singapore Institute for Clinical Sciences, Agency for Science, Technology and Research, Singapore, Singapore; 8 Department of Obstetrics & Gynaecology, Yong Loo Lin School of Medicine, National University of Singapore, Singapore, Singapore; 9 Department of Paediatrics, Yong Loo Lin School of Medicine, National University of Singapore, Singapore; 10 Khoo Teck Puat-National University Children’s Medical Institute, National University Hospital, National University Health System, Singapore, Singapore; 11 Center for Quantitative Medicine, Duke-NUS Medical School, Singapore, Singapore; 12 Tampere Center for Child Health Research, University of Tampere and Tampere University Hospital, Tampere, Finland; 13 Department of Paediatrics, KK Women’s and Children’s Hospital, Singapore, Singapore; 14 Lee Kong Chian School of Medicine, Nanyang Technological University, Singapore, Singapore; 15 Yong Loo Lin School of Medicine, National University of Singapore, Singapore; 16 Family Medicine Service, KK Women’s and Children’s Hospital, Singapore, Singapore

## Abstract

**Importance:**

Global cesarean delivery (CD) rates have more than doubled over the past 2 decades, with an increasing contribution from elective CDs. Cesarean delivery has been linked to early childhood overweight and obesity, but limited studies have examined elective and emergency CDs separately.

**Objective:**

To investigate whether elective or emergency CD was associated with risk of early childhood overweight.

**Design, Setting, and Participants:**

Data were drawn from the Growing Up in Singapore Toward Healthy Outcomes (GUSTO) study, an ongoing prospective mother-child birth cohort study. Participants were pregnant women aged 18 years or older with homogeneous parental ethnic background in their first trimester recruited between June 2009 and September 2010 (n = 1237) at 2 major public hospitals in Singapore. Those with type 1 diabetes or undergoing chemotherapy or psychotropic drug treatment were excluded. Data analysis commenced in October 2017.

**Exposures:**

Delivery mode obtained from clinical records. Elective and emergency CD examined separately against vaginal delivery as reference.

**Main Outcomes and Measures:**

Body mass index–for–age *z* scores at age 12 months calculated based on 2006 World Health Organization Child Growth Standards from infant weight and recumbent crown-heel length measurements taken between December 2010 and April 2012. High body mass index status *at risk of overweight* was defined as a *z* score of more than 1 SD and less than or equal to 2 SDs. *Overweight* was defined as a *z* score of more than 2 SDs.

**Results:**

Among 727 infants analyzed (51.2% [372] male), 30.5% (222) were born via CD, of which 33.3% (74) were elective. Prevalence of at risk of overweight and overweight at age 12 months was 12.2% (89) and 2.3% (17), respectively. Elective CD was significantly associated with at risk of overweight or overweight at age 12 months after adjusting for maternal ethnicity, age, education, parity, body mass index, antenatal smoking, hypertensive disorders of pregnancy, gestational diabetes, and sex-adjusted birth weight–for–gestational age (odds ratio, 2.05; 95% CI, 1.08-3.90; *P* = .03). The association persisted after further adjustment for intrapartum antibiotics and first 6 months infant feeding, 2 potential mediators of early childhood overweight and obesity (odds ratio, 2.02; 95% CI, 1.05-3.89; *P* = .04). No significant associations were found for emergency CD. Analysis with multiple imputation for missing covariates yielded similar results.

**Conclusions and Relevance:**

Choice of delivery mode may influence risk of early childhood overweight. Clinicians are encouraged to discuss potential long-term implications of elective CD on child metabolic outcomes with patients who intend to have children.

## Key Points

**Question:**

Are elective and emergency cesarean delivery both associated with risk of childhood overweight at age 12 months?

**Findings:**

In this cohort study that analyzed 727 mother-child pairs, elective cesarean delivery was significantly associated with high body mass index–for–age *z* score at 12 months. Emergency cesarean delivery was not significantly associated with high body mass index–for–age *z* score at 12 months.

**Meaning:**

Choice of delivery mode may influence risk of early childhood overweight, which is a concern clinicians may discuss with patients who intend to have children.

## Teaser

This cohort study uses data from the Growing Up in Singapore Toward Healthy Outcomes study to investigate the association between elective and emergency cesarean delivery and early childhood overweight at 12 months.

## Introduction

Global cesarean delivery (CD) rates have more than doubled over the past 2 decades, particularly in more developed countries.^1^ Noteworthily, the contribution from elective CD has increased significantly.^2,3,4^ Elective CDs are typically performed in the absence of maternal or fetal indications.^5^ The rising CD rates have been attributed to multiple biopsychosocioeconomic and cultural factors, including increased maternal age, increased prevalence of gestational diabetes mellitus (GDM), fear of childbirth, favorable public perception, and increased consumer autonomy, of which different permutations are likely to exist among populations.^6,7,8^

Cesarean delivery has been associated with early childhood overweight and obesity amidst inconclusive evidence.^9,10,11,12,13^ Widely regarded as a public health epidemic, overweight and obesity are linked to childhood and adult morbidities such as cardiovascular disease, type 2 diabetes, orthopedic problems, depression, low self-esteem, and social marginalization.^14,15,16^ Early childhood overweight and obesity are also likely to persist into middle childhood and adolescence.^17,18^ In 2016, the World Health Organization (WHO) estimated that 41 million children younger than 5 years were affected by overweight or obesity worldwide, half of them in Asia.^19^ The same report highlighted the importance of addressing obesogenic risk factors in pregnancy and infancy, 2 critical developmental periods, with delivery mode implicated as 1 such factor.

Various schools of thought underlie the association between delivery mode and early childhood overweight and obesity. Studies have shown that delivery mode establishes initial gut microbiome diversity in newborns,^20^ which continues to diverge throughout infancy until age 6 months.^21,22,23^ This led to the theory that the altered initial gut microbiome in infants born via CD modulates energy harvest and metabolism through interaction with diet, thereby increasing susceptibility to subsequent overweight.^24,25^ Proponents of the microbiota maturity concept, however, believe that time point of acquisition of a mature gut microbiota, rather than initial microbiome, was predictive of subsequent adiposity.^26,27^ Alternatively, it has been proposed that stress-response signaling during delivery may shape long-term metabolic trajectories via epigenetics^28^ or hypothalamic-pituitary-adrenal axis modulation independent of microbiome.^29^ Although the exact underlying mechanism remains unclear, we hypothesize that infants born via elective CD differ from those born via emergency CD in terms of overweight susceptibilities, likely inherently due to absence or presence of labor or membrane rupture. However, few studies have examined emergency and elective CD separately owing to inadequate data on delivery mode subgroups, which may partially explain the inconclusive evidence of association.^30,31,32^

Variable study setting and covariate adjustment may also contribute to the contradictory results. Results from existing population-specific studies may have limited generalizability owing to strong racial/ethnic and geographical disparities in childhood overweight and obesity.^33,34^ Gestational diabetes, prepregnancy body mass index (BMI [calculated as weight in kilograms divided by height in meters squared]), maternal and infant antibiotics, and birth weight, together with gestational age, were common confounders not adjusted for in studies investigating the association between cesarean delivery and childhood overweight and obesity.^35,36^ With increasingly multiethnic communities due to enhanced global migration and mobility, more comprehensive population-specific prospective studies on the impact of elective and emergency CD on early childhood overweight and obesity are warranted.

Controlling for potential confounding and mediating effects from multiple maternal and infant factors, this mother-offspring birth cohort study of a multiethnic Asian population aimed to investigate whether elective and emergency CD were independently associated with risk of childhood overweight at age 12 months. We hypothesize that infants born via elective CD vs emergency CD may differ in risk of overweight during early childhood.

## Methods

### Study Design

Data were drawn from the ongoing Growing Up in Singapore Toward Healthy Outcomes (GUSTO) prospective mother-child birth cohort study.^37^ Data analysis commenced in October 2017. Pregnant women in their first trimester (n = 1237) were recruited between June 2009 and September 2010 from KK Women’s and Children’s Hospital or National University Hospital, 2 major public hospitals in Singapore. Citizens or permanent residents aged 18 years or older with homogeneous parental ethnic background (Chinese, Malay, or Indian) were included. Those with type 1 diabetes or undergoing chemotherapy or psychotropic drug treatment were excluded.

All participants gave written informed consent. Ethical approval was obtained from the Centralised Institutional Review Board of SingHealth and Domain Specific Review Board of Singapore National Health Care Group. This study follows the Strengthening the Reporting of Observational Studies in Epidemiology (STROBE) reporting guideline for cohort studies.

### Exposures

Delivery mode was obtained from hospital delivery reports by trained health personnel. Vaginal delivery included spontaneous vaginal delivery, assisted breech delivery, vacuum extraction, and forceps delivery. For this study, elective CD was defined as a CD conducted as a result of advanced planning due to reasons such as maternal request, history of CD and malpresentation, and/or a decision made more than 24 hours before delivery due to nonemergency maternal or fetal conditions such as maternal obesity, diabetes, and macrosomia. Emergency CD was defined as a CD that was not planned or scheduled, and/or a decision made during the 24 hours before delivery due to deteriorating maternal or fetal health. These are working definitions by health care professionals in practice. To better address our hypothesis, CD was reclassified into intrapartum and nonlabor according to whether the procedure took place before or after labor onset and/or membrane rupture.

### Anthropometric Outcome

Between December 2010 and April 2012, infant anthropometric measurements were taken by trained health personnel at age 12 months. Weight was measured to the nearest 0.001 kg using the Seca 334 calibrated weighing scale (Seca GmbH & Co KG). Recumbent crown-heel length was measured to the nearest 0.1 cm using a Seca 210 Mobile Measuring Mat.

Based on WHO 2006 Child Growth Standards, infant weight and length were computed into non–ethnic-specific BMI-for-age *z* scores (BAZs) using WHO Anthro software (version 3.2.2).^38^ *At risk of overweight* was defined as a BAZ greater than 1 SD and less than or equal to 2 SDs and *overweight* was defined as a BAZ greater than 2 SDs.^39^ These high BMI statuses^40^ were analyzed as a single category in this study. *Not at risk of overweight* was defined as a BAZ less than or equal to 1 SD.

### Covariates

Maternal ethnicity, age, educational level, antenatal smoking status, and infant feeding mode during first 6 months of life were assessed through questionnaires administered by interviewers who were trained health personnel. Early pregnancy BMI, hypertensive disorders of pregnancy, intrapartum antibiotic administration, and infant sex, birth weight, and gestational age were obtained from hospital case notes. Sex-adjusted birth weight–for–gestational age (BW-for-GA) *z* scores were subsequently calculated.^41^ Early pregnancy BMI was classified according to the WHO reference for Asian individuals^42^ and was preferred to prepregnancy BMI because of concerns of recall bias and underreporting of prepregnancy weight, especially for the higher BMI groups.^43,44^ A high correlation was found between the 2 variables in this study sample (Pearson correlation coefficient *r* = 0.96; *P* < .001). Gestational diabetes was diagnosed based on WHO criteria.^45^ Details are included in the eAppendix in the Supplement.

### Statistical Analysis

Participant characteristics were compared using χ^2^ or Fisher exact tests for categorical variables and 1-factor analysis of variance for continuous variables (eAppendix in the Supplement).

Association of delivery mode with at risk of overweight and overweight at age 12 months was analyzed using binary logistic regression (n = 727). Three models were used. Model 1 gave the unadjusted association (crude model). Model 2 was adjusted for potential confounders, including maternal ethnicity, age at delivery (continuous), educational level, parity, early pregnancy BMI, antenatal active or passive smoking, hypertensive disorders of pregnancy, GDM, and infant sex-adjusted BW-for-GA *z* score (continuous) (eAppendix in the Supplement). Continuous covariates were used in linear terms. We used linktest to check whether the nonlinear form of a continuous variable provided any better fit than the linear form. Model 3 further adjusted for intrapartum antibiotics and infant feeding during the first 6 months, 2 potential mediators of early childhood overweight. In each of these models, CD subgroups were analyzed as 2 indicator variables with vaginal delivery as reference. Potential interactions between covariates and delivery mode were tested using cross-product terms.

Secondary analyses were repeated using multiply imputed missing values for covariates in regression analysis. Maternal education (n = 10), early pregnancy BMI (n = 58), antenatal smoking status (n = 11), GDM (n = 65), intrapartum antibiotics (n = 1), sex-adjusted BW-for-GA *z* score (n = 5), and infant feeding (n = 115) were imputed 20 times via multiple imputation analyses by chained equation^46^ such that the total sample size was 956. Results of the 20 analyses were pooled using the Rubin rule^47^ (eAppendix in the Supplement).

Additional association analyses were performed with the outcome variables of BAZ in continuous form and BMI status in ordinal form using multiple linear regression and ordinal logistic regression (Probit link), respectively.

Maternal and infant characteristics are presented as number (percentage) or mean (SD). Regression analyses are presented as odds ratios (ORs) or β coefficients with 95% confidence intervals. Two-tailed *P* < .05 was considered statistically significant. Statistical analyses were performed using SPSS Statistics version 23.0 (IBM) and Stata release 13 (StataCorp). Syntax is available in the eAppendix in the Supplement.

## Results

### Study Participants

Of the 1237 mother-child pairs recruited, 727 (51.2% [372] male infants) were included in the primary analysis (Figure). The 3 subsets of participants had similar maternal education, early pregnancy BMI, hypertensive disorders, intrapartum antibiotics, delivery mode, infant sex, infant feeding during first 6 months, and BAZ at age 12 months, but differed in terms of ethnicity, age, parity, antenatal smoking status, GDM, and sex-adjusted BW-for-GA *z* score (eTable 1 in the Supplement).

**Figure.**
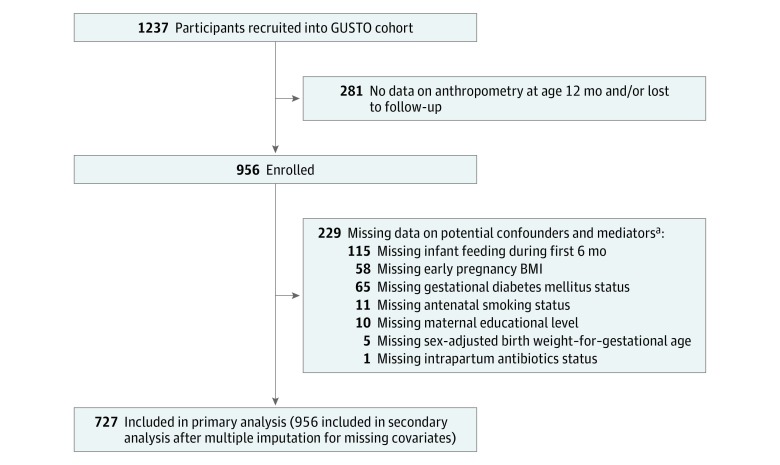
Flowchart of the Study Population BMI indicates body mass index (calculated as weight in kilograms divided by height in meters squared); GUSTO, Growing Up in Singapore Toward Healthy Outcomes. ^a^Some participants missing more than 1 covariate.

Of the 727 singletons analyzed, 30.5% (222) were born via CD. Elective CD rate was 10.2%, or 33.3% (74) of total CD births. The nonlabor CD rate was 19.8%, or 64.9% (144) of total CD births. Prevalence of at risk of overweight and overweight at age 12 months was 12.2% (89) and 2.3% (17), respectively.

Maternal and infant characteristics among the different modes of delivery are summarized in Table 1. Infants born via elective CD, compared with vaginally delivered infants, had the highest sex-adjusted BW-for-GA *z* score (0.44 vs 0.11) and BAZ at age 12 months (0.13 vs −0.19) and were more likely to be born to mothers of older age (33.10 vs 31.19 years) and higher parity (82.4% vs 60.6%). Infants born via emergency CD were more likely than infants born via elective CD to be born to mothers who had hypertensive disorders of pregnancy (11.5% vs 5.5%) and intrapartum antibiotics administered (48.6% vs 32.3%). Row percentages are presented in eTable 2 in the Supplement.

**Table 1.** Comparison of Maternal and Infant Characteristics Among Different Modes of Delivery

Variable	No. (%)			*P* Value^a^
	Vaginal Delivery (n = 505)	Cesarean Delivery		
		Emergency (n = 148)	Elective (n = 74)	
**Maternal**				
Ethnicity				
Chinese	293 (58.0)	76 (51.4)	42 (56.8)	.63
Malay	127 (25.1)	42 (28.4)	17 (23.0)	
Indian	85 (16.8)	30 (20.3)	15 (20.3)	
Maternal age at delivery, mean (SD) [range], y	31.19 (5.03) [18.9-44.5]	31.05 (5.44) [19.6-42.9]	33.10 (5.12) [20.9-46.9]	.008
Maternal education				
No formal education or primary or secondary education only	151 (29.9)	44 (29.7)	21 (28.4)	.62
Postsecondary	173 (34.3)	60 (40.5)	26 (35.1)	
University	181 (35.8)	44 (29.7)	27 (36.5)	
Parity				
0	199 (39.4)	91 (61.5)	13 (17.6)	<.001
≥1	306 (60.6)	57 (38.5)	61 (82.4)	
Early pregnancy BMI status				
Underweight, BMI <18.5	48 (9.5)	7 (4.7)	5 (6.8)	.14
Increasing but acceptable risk, BMI ≥18.5 and <23	239 (47.3)	72 (48.6)	28 (37.8)	
Increased risk, BMI ≥23 and <27.5	138 (27.3)	38 (25.7)	22 (29.7)	
High risk, BMI ≥27.5	80 (15.8)	31 (20.9)	19 (25.7)	
Active or passive smoking during pregnancy				
No	290 (57.4)	73 (49.3)	44 (59.5)	.18
Yes	215 (42.6)	75 (50.7)	30 (40.5)	
Hypertensive disorders of pregnancy				
No	477 (94.5)	131 (88.5)	73 (98.6)	.006
Yes	28 (5.5)	17 (11.5)	1 (1.4)	
Gestational diabetes mellitus				
No	423 (83.8)	120 (81.1)	59 (79.7)	.57
Yes	82 (16.2)	28 (18.9)	15 (20.3)	
Intrapartum antibiotics				
No	342 (67.7)	76 (51.4)	66 (89.2)	<.001
Yes	163 (32.3)	72 (48.6)	8 (10.8)	
**Infant**				
Sex				
Male	260 (51.5)	80 (54.1)	32 (43.2)	.31
Female	245 (48.5)	68 (45.9)	42 (56.8)	
Sex-adjusted birth weight–for–gestational age *z* score, mean (SD) [range]	0.11 (1.12) [−2.83 to 3.56]	0.27 (1.39) [−3.07 to 8.65]	0.44 (1.33) [−2.96 to 3.96]	.046
Feeding during first 6 mo				
Exclusive breastfeeding	73 (14.5)	12 (8.1)	11 (14.9)	.16
Mixed feeding	318 (63.0)	91 (61.5)	46 (62.2)	
Exclusive formula feeding	114 (22.6)	45 (30.4)	17 (23.0)	
BAZ at 12 mo, mean (SD) [range]	−0.19 (1.00) [−3.62 to 2.65]	−0.19 (1.08) [−2.76 to 2.38]	0.13 (1.11) [−2.78 to 2.22]	.04
BMI status at 12 mo				
Not at risk of overweight, BAZ ≤1 SD	439 (86.9)	126 (85.1)	56 (75.7)	.12
At risk of overweight, BAZ >1 SD and ≤2 SDs	55 (10.9)	18 (12.2)	16 (21.6)	
Overweight, BAZ >2 SD	11 (2.2)	4 (2.7)	2 (2.7)	

Abbreviations: BAZ, body mass index–for–age *z* score; BMI, body mass index (calculated as weight in kilograms divided by height in meters squared).

^a^ χ^2^ or Fisher exact tests were used for categorical variables and 1-factor analysis of variance was used for continuous variables to compare the 3 groups.

### Delivery Mode and Anthropometric Outcomes

Elective CD was significantly associated with risk of overweight and overweight at age 12 months (unadjusted OR, 2.14; 95% CI, 1.18-3.86; *P* = .01) (Table 2). The association remained significant after adjusting for potential confounding effects from maternal ethnicity, age at delivery, educational level, parity, early pregnancy BMI, antenatal active or passive smoking, hypertensive disorders of pregnancy, GDM, and infant sex-adjusted BW-for-GA (adjusted OR, 2.05; 95% CI, 1.08-3.90; *P* = .03). The association persisted after additional adjustment for intrapartum antibiotics and infant feeding during the first 6 months, 2 potential mediators of early childhood overweight and obesity (final adjusted OR, 2.02; 95% CI, 1.05-3.89; *P* = .04). No significant associations were found for emergency CD in unadjusted and adjusted models.

**Table 2.** Association of Delivery Mode With Risk of Overweight and Overweight at Age 12 Months for 727 Participants^a^

Delivery Mode	Model 1^b^		Model 2^b^		Model 3^b^	
	OR (95% CI)	*P* Value	OR (95% CI)	*P* Value	OR (95% CI)	*P* Value
Vaginal	1 [Reference]		1 [Reference]		1 [Reference]	
Emergency cesarean	1.16 (0.69-1.96)	.57	0.93 (0.53-1.62)	.79	0.95 (0.54-1.68)	.86
Elective cesarean	2.14 (1.18-3.86)	.01	2.05 (1.08-3.90)	.03	2.02 (1.05-3.89)	.04

Abbreviation: OR, odds ratio.

^a^ Data were analyzed using logistic regression.

^b^ Model 1 was unadjusted. Model 2 was adjusted for ethnicity, maternal age at delivery, maternal educational level, parity, early pregnancy body mass index, antenatal active or passive smoking, hypertensive disorders of pregnancy, gestational diabetes, and sex-adjusted birth weight–for–gestational age *z* score. Model 3 was adjusted for all variables from model 2 as well as intrapartum antibiotics and infant feeding during the first 6 months.

Regression analyses with imputed missing covariates returned similar results, showing elective CD significantly associated with risk of overweight and overweight at age 12 months (unadjusted OR, 2.13; 95% CI, 1.25-3.62; *P* = .005 and final adjusted OR, 1.93; 95% CI, 1.07-3.48; *P* = .03), but no association for emergency CD (Table 3).

**Table 3.** Association of Delivery Mode With Risk of Overweight and Overweight at Age 12 Months After Multiple Imputation for Missing Covariates in 956 Participants^a^

Delivery Mode	Model 1^b^		Model 2^b^		Model 3^b^	
	OR (95% CI)	*P* Value	OR (95% CI)	*P* Value	OR (95% CI)	*P* Value
Vaginal	1 [Reference]		1 [Reference]		1 [Reference]	
Emergency cesarean	1.30 (0.82-2.04)	.26	1.08 (0.66-1.76)	.76	1.15 (0.70-1.89)	.58
Elective cesarean	2.13 (1.25-3.62)	.005	2.01(1.13-3.58)	.02	1.93 (1.07-3.48)	.03

Abbreviation: OR, odds ratio.

^a^ Data were analyzed using logistic regression.

^b^ Model 1 was unadjusted. Model 2 was adjusted for ethnicity, maternal age at delivery, maternal educational level, parity, early pregnancy body mass index, antenatal active or passive smoking, hypertensive disorders of pregnancy, gestational diabetes, and sex-adjusted birth weight–for–gestational age *z* score. Model 3 was adjusted for all variables from model 2 as well as intrapartum antibiotics and infant feeding during the first 6 months.

Additional analyses using reclassified CD yielded similar trends of association. Nonlabor CD was associated with risk of overweight and overweight at age 12 months (unadjusted OR, 1.83; 95% CI, 1.14-2.93; *P* = .01) (eTable 3 in the Supplement). Associations were not significant in adjusted models (*P* = .06).

Analyses using BAZ as a continuous outcome variable and BMI status as an ordinal outcome variable (ie, not at risk of overweight, at risk overweight, overweight) produced findings consistent with our primary analyses (eTables 4 and 5 in the Supplement).

Sensitivity analyses stratified by parity were performed, as significant interaction was found for parity. Both CD modes were significantly associated with risk of overweight and overweight among parous mothers, with elective CD having a higher OR (eTable 6 in the Supplement). Among nulliparous mothers, only emergency CD was significantly associated with lower risk of overweight despite similar ORs.

## Discussion

Our study provides novel evidence of an association between elective CD and increased childhood overweight risk as early as the age of 12 months. The few existing studies we are aware of that examined CD subgroups separately and similarly found an increased risk for elective CD included older children aged 3 to 7 years.^30,31,32^ Among studies that did not discriminate between elective and emergency CD, the earliest age of proven association was 2 years.^13^ These studies also had more mixed results, with a few finding no significant association between CD and early childhood overweight.^13,48,49^ Future studies may consider analyzing CD subgroups separately to increase clarity of results. With early infancy implicated as a critical obesogenic microbiome developmental period, we postulate that earlier outcome assessment may yield greater effect of association due to lesser interference from external influences, such as diet, sleep, and physical activity. Assessment at an earlier time may also reduce sample attrition rates in prospective studies, increasing sample size and thus statistical power.

Our main findings revealed disparate association of elective and emergency CD with early childhood overweight. Additional analyses demonstrated a greater risk in nonlabor CD compared with intrapartum CD. Together, these findings substantiate our hypothesis that infants born via elective CD differ from infants born via emergency CD in terms of overweight susceptibilities, likely owing to absence of labor exposure and/or membrane rupture. One possible underlying mechanism could be the partial microbial exposure during emergency CD from membrane rupture, as proposed by a 2013 study^50^ that found the lowest bacterial richness and diversity in fecal microbiota of infants born via elective CD at age 3 to 4 months. Alternatively, it could be that the substantially lower stress response from absence of labor stimulus experienced by infants born via elective CD, shown to have the lowest serum cortisol levels at birth,^51^ conferred a suboptimal differentiation of cell types involved in long-term health.^52^ An interplay of these factors with the environment may be more likely.

However, our main findings are contrary to existing studies that unanimously found an association between nonelective CD and early childhood overweight.^30,31,32^ A recent study also reported higher abundance of proadipogenic Lachnospiraceae family and higher risk of overweight and obesity in infants born via emergency CD to obese mothers.^53^ This obesogenic vertical microbiome transmission theory is supported by evidence of independent association of maternal overweight and obesity with neonatal gut microbiome and childhood overweight and obesity, albeit in infants born via CD not classified as emergency or elective.^54^ The conflicting results may have arisen from varying definitions of elective CD (participant-reported elective vs maternal request vs planned CD) and lack of adjustment for intrapartum antibiotics, an important mediating factor. Another reason may be residual confounding, a challenge in studies examining causality between delivery mode and childhood overweight and obesity. Some studies attempted to circumvent this by investigating sibling discordance, but results remained inconclusive.^55,56^

In addition, our sensitivity analyses provided new evidence of parity as a modifying factor. Opposite trends of association between emergency CD and risk of early childhood overweight were observed for nulliparous and parous women. This was similar for elective CD, although not achieving statistical significance in nulliparous women. However, it must be noted that the small sample size (n = 13) of elective CD in the nulliparous subgroup could have restricted statistical power. Existing literature inconsistently adjusted for parity, and no studies stratified their analyses by parity, to our knowledge.^30,31,32,48,49,55,56^ Hence, we can only postulate that our findings on parity may be a consequence of the association between multiparity and maternal overweight and obesity,^57^ which then parallels the obesogenic vertical microbiome transmission theory discussed above, although again, parity was not adjusted for in these studies.^53,54^ Further studies are needed to prove or disprove this. Nevertheless, our study highlights the importance of (1) analyzing CD subgroups separately and (2) stratifying analyses by parity in gaining better understanding of the relationship between delivery mode and early childhood overweight.

The overall CD rate of 30.5% in our study is comparable to that of 30.5% published by the Ministry of Health, Singapore, in 2004, although the breakdown of CD subgroups was not reported then.^58^ It is, however, higher than the Southeast Asia estimate of 14.8% and the estimate of 27.2% for more developed countries published by a recent worldwide analysis, although still within the reported ranges of 1.7% to 32.0% and 13.9% to 38.1%, respectively.^1^ This is worrisome, as overall CD rates above the threshold of 9% to 16% were reportedly not associated with decreases in maternal, neonatal, and infant mortality outcomes even after adjusting for socioeconomic factors.^59^ The procedure has been associated with poorer short- and long-term maternal outcomes, including longer hospital stay and placenta previa, and increased risks of undesirable neonatal and childhood health outcomes, including respiratory complications, atopy, allergy, and type 1 diabetes.^7,60,61,62,63^

Existing studies on CD subgroup rates, however, were far less common. Elective and emergency CD rates in our study were 10.2% and 20.3%, while nonlabor and intrapartum CD rates were 19.8% and 10.7%, respectively. As past figures were not available, we were unable to assess the local elective CD trend. A 2009 Southeast Asian study^64^ found that maternal request contributed 2.1% to the overall CD rate in Indonesia. Our results were closer to estimates in developed countries. The 2010 UK national estimates were 9.3% and 14.5% for elective and emergency CD, respectively, defined based on the UK Office for Population Censuses and Surveys classification codes R17 (not in labor) and R18 (in labor).^65^ Switzerland estimated the rate of “caesarean section on maternal request prior to the onset of labour”^3^ at 5.1% and “caesarean section with medical indication prior to and after onset of labour”^3^ at 22.3%. It is apparent that elective CD is loosely defined among studies, with some considering it an indication. The elective CD rate in our study increased substantially when it was reclassified as nonlabor CD, further emphasizing the point that CD definitions are a crucial aspect of future studies. Therefore, there is a pressing need for greater specificity and standardization of CD subgroups to yield more comparable results and better insights.

Prevalence of at risk of overweight in our study was 12.2%, compared with the Southeast Asia estimate of 8.1% and the estimate of 21.4% for developed countries.^66^ These estimates were based on children aged 0 to 5 years using similar WHO Child Growth Standards cutoffs. Prevalence of overweight in our study was 2.3%, compared with the 4.6% and 11.7% estimates for Southeast Asia and developed countries, respectively.^66^ A Singapore study on Chinese preschoolers aged 6 to 72 months found an overweight prevalence of 7.5% to 8.1% using 3 different BMI-based cutoffs.^67^ The variation in results may be due to different age groups examined and different cutoffs defining childhood weight status. The high prevalence of at risk of overweight is a cause for concern, especially as worldwide rates have been projected to increase by 2020.^68^ From a public health perspective, there may be value in allocating resources for preventive efforts targeting at-risk infants.

Strengths of our study included large sample size, longitudinal design, and standardization of data collection, which helped increase reliability and validity. Data collection was designed to minimize recall bias; participants were not required to recall prepregnancy BMI in favor of pregnancy BMI, and infant feeding was assessed at 3-month instead of 6-month intervals. The comprehensive data also allowed for examination of CD subgroups separately while accounting for important potential confounding and mediating factors such as GDM and intrapartum antibiotics. Intrapartum antibiotics had not been adjusted for in previous studies. Antibiotics administered during labor have been shown to affect vertical transmission of maternal vaginal microflora, and thus should not be ignored as an important mediating factor.^69^

### Limitations

A limitation of our study was the lack of data on paternal characteristics. Studies have proven paternal overweight and obesity and its underlying genetic and environmental basis, including familial predisposition to obesogenic appetitive traits, paternal eating behavior, and parenting style, as a significant risk factor of childhood overweight.^70,71,72,73,74^ Adjustment for paternal factors would have contributed to increased study robustness. In addition, our comparison analyses suggest potential selection bias in the primary analysis group. However, the differing characteristics have been adjusted for as covariates in the regression analyses, so they are unlikely to have caused bias in the results. Furthermore, secondary analyses that included 229 additional participants returned similar results. Measurement errors may also potentially exist, although the same calibrated equipment was used by trained health personnel and the average of multiple measurements were taken. The multiethnicity of our study population, while giving us insight into delivery mode and early childhood overweight in a local context, may limit the generalizability of our results.

## Conclusions

This study suggests that choice of delivery mode may influence risk of early childhood overweight. More population-specific prospective studies examining emergency and elective CD as separate subgroups are warranted. Additional studies may explore population-specific factors underlying the rising trend in elective CD rates. Further validation of these findings may expand our preventive strategy against childhood metabolic disorders. Clinicians may be encouraged to discuss potential long-term implications of elective CD on child metabolic outcomes with patients who intend to have children.

## Notes

Supplement. **eAppendix.** Detailed Methods **eTable 1.** Comparison of Maternal and Infant Characteristics Among Three Subsets of Participants **eTable 2.** Row Percentages of Categorical Variables Compared Among Different Modes of Delivery **eTable 3.** Association of Intrapartum and Non-Labour Caesarean Delivery With Risk of Overweight/Overweight at Age 12 Months **eTable 4.** Association of Delivery Mode With Continuous Outcome Variable BMI z-score (BAZ) at Age 12 Months **eTable 5.** Association of Delivery Mode With BMI Status at Age 12 Months in Ordinal Form **eTable 6.** Association of Delivery Mode With Risk of Overweight/Overweight at Age 12 Months Stratified According to Parity **eReferences**
