# Supplementary material for: Association of Elective and Emergency Cesarean Delivery With Early Childhood Overweight at 12 Months of Age
Source: JAMA Netw Open. 2018 Nov 21;1(7):e185025. doi: 10.1001/jamanetworkopen.2018.5025 (PMC6324378; doi:10.1001/jamanetworkopen.2018.5025)
Supplement: Supplement. — eAppendix. Detailed Methods eTable 1. Comparison of Maternal and Infant Characteristics Among Three Subsets of Participants eTable 2. Row Percentages of Categorical Variables Compared Among Different Modes of Delivery eTable 3. Association of Intrapartum and Non-Labour Caesarean Delivery With Risk of Overweight/Overweight at Age 12 Months eTable 4. Association of Delivery Mode With Continuous Outcome Variable BMI z-score (BAZ) at Age 12 Months eTable 5. Association of Delivery Mode With BMI Status at Age 12 Months in Ordinal Form eTable 6. Association of Delivery Mode With Risk of Overweight/Overweight at Age 12 Months Stratified According to Parity eReferences [file jamanetwopen-1-e185025-s001.pdf]

Supplementary Online Content

Cai M, Loy SL, Tan KH, et al. Association of elective and emergency cesarean delivery with early childhood overweight at 12 months of age. *JAMA Netw Open*. 2018;1(7):e185025. doi:10.1001/jamanetworkopen.2018.5025

eAppendix. Detailed Methods

eTable 1. Comparison of Maternal and Infant Characteristics Among Three Subsets of Participants

eTable 2. Row Percentages of Categorical Variables Compared Among Different Modes of Delivery

eTable 3. Association of Intrapartum and Non-Labour Caesarean Delivery With Risk of Overweight/Overweight at Age 12 Months

eTable 4. Association of Delivery Mode With Continuous Outcome Variable BMI z-score (BAZ) at Age 12 Months

eTable 5. Association of Delivery Mode With BMI Status at Age 12 Months in Ordinal Form

eTable 6. Association of Delivery Mode With Risk of Overweight/Overweight at Age 12 Months Stratified According to Parity

eReferences

This supplementary material has been provided by the authors to give readers additional information about their work.

© 2018 Cai M et al. *JAMA Network Open.*

eAppendix. Detailed methods.

Covariates in regression models

Maternal ethnicity, age, and educational level were collected through interviewer-administered questionnaires by trained health personnel at recruitment. Antenatal active and/or passive smoking status was similarly assessed at 26-28 weeks gestation with the following questions: “Are you currently smoking?”, “Anyone living at home who smokes daily?”, and “Are you currently exposed to cigarette smoke daily?”. Infant feeding mode during first 6 months of life (exclusive breastfeeding, partial breastfeeding, or exclusive formula feeding) was assessed with feeding questionnaires at age 3 and 6 months. Early pregnancy BMI, calculated from maternal weight at ≤14 weeks gestation obtained from hospital case notes and maternal height measured with a Seca 213 stadiometer (Seca) at 26-28 weeks gestation, was classified according to WHO reference for Asians: underweight (<18.5 kg/m^2), increasing but acceptable risk (18.5 ≤kg/m^2 <23), increased risk (23 ≤kg/m^2 <27.5), and high risk (≥27.5 kg/m^2)[1]. Hypertensive disorders of pregnancy included chronic hypertension with superimposed preeclampsia, pregnancy-induced hypertension, preeclampsia and eclampsia. GDM was diagnosed based on WHO criteria of fasting plasma glucose ≥7 mmol/L and/or 2 h post-load concentration ≥7.8 mmol/L following 75 g oral glucose tolerance tests at 26-28 weeks gestation[2].

Participant characteristics comparison statistics

Chi-Square and Fisher’s Exact tests: For categorical variable “BMI status at age 12 months” in Table 1, 2 cells (22.2%) had an expected count of less than 5 and the minimum expected count was 1.73. Hence Fisher’s Exact test was used in the comparison between the three delivery modes (test statistic = 6.98). Chi-Square tests were used for all other categorical variables as assumptions of ≥1 expected count for each cell and ≥5 expected count for majority (80%) of cells were met: “maternal ethnicity” (Chi-Square statistic χ^2 = 2.61), “maternal education” (χ^2 = 2.64), “parity” (χ^2 = 42.66), “early pregnancy BMI status” (χ^2 = 9.56), “active/passive smoking during pregnancy” (χ^2 = 3.45), “hypertensive disorders of pregnancy” (χ^2 = 10.26), “GDM” (χ^2 = 1.13), “intrapartum antibiotics” (χ^2 = 32.72), “infant sex” (χ^2 = 2.37) and “infant feeding during first 6 months” (χ^2 = 6.66). Similarly, Chi-Square tests were used for all categorical variables in Supplementary eTable 1: “maternal ethnicity” (Chi-Square statistic χ^2 = 14.53), “maternal education” (χ^2 = 20.98), “parity” (χ^2 = 14.36), “early pregnancy BMI status” (χ^2 = 3.18), “active/passive smoking during pregnancy” (χ^2 = 8.81), “hypertensive disorders of pregnancy” (χ^2 = 1.39), “GDM” (χ^2 = 6.24), “intrapartum antibiotics” (χ^2 = 0.03), “delivery mode” (χ^2 = 2.64), “reclassified delivery mode” (χ^2 = 2.67), “infant sex” (χ^2 = 2.41) and “infant feeding during first 6 months” (χ^2 = 6.10).

One-factor analysis of variance (ANOVA): The following continuous variables were compared between the three delivery modes in Table 1: “maternal age at delivery” (F-statistic F = 4.80), “sex-adjusted birth weight-for-gestational age z-score” (F = 3.10) and “BMI-for-age z-score (BAZ) at age 12 months” (F = 3.21). Continuous variables were similarly compared between the three participant subsets in Supplementary eTable 1: “maternal age at delivery” (F-statistic F = 7.84), “sex-adjusted birth weight-for-gestational age z-score” (F = 7.41) and “BMI-for-age z-score (BAZ) at age 12 months” (F = 1.09).

Logistic regression model

Logistic regression analysis began with an unadjusted model (model 1) examining crude association between delivery mode [categorical: normal vaginal delivery (coded “0”), emergency caesarean delivery (coded “1”) or elective caesarean delivery (coded “2”)] and early childhood overweight [categorical: not at risk of overweight (coded “0”) or at risk of overweight/overweight (coded “1”)]. Model 2 included adjustment for the following potential confounders: maternal ethnicity [categorical: Chinese (coded “0”), Malay (coded “1”) or Indian (coded “2”)], age at delivery (continuous), educational level [categorical: no-formal/primary/secondary (coded “0”), post-secondary (coded “1”) or university (coded “2”)], parity [(categorical: 0 (coded “0”) or ≥ 1 (coded “1”)), early pregnancy BMI [categorical: underweight (<18.5 kg/m^2; coded “0”), increasing but acceptable risk (18.5 ≤kg/m^2 <23; coded “1”), increased risk (23 ≤kg/m^2 <27.5; coded “2”), or high risk (≥27.5 kg/m^2; coded “3”)], antenatal active/passive smoking [categorical: no (coded “0”) or yes (coded “1”)], hypertensive disorders of

© 2018 Cai M et al. *JAMA Network Open.*

pregnancy [categorical: no (coded "0") or yes (coded "1")], GDM [categorical: no (coded "0") or yes (coded "1")], and infant sex-adjusted BW-for-GA z-score (continuous). Model 3 included additional adjustment for potential mediating factors: intrapartum antibiotics [categorical: no (coded "0") or yes (coded "1")] and infant feeding during first 6 months of life [categorical: exclusive breastfeeding (coded "0"), mixed feeding (coded "1") or exclusive formula feeding (coded "2")]. The reference group for each dependent variable was that coded as "0". Logistic regression was run with classification cutoff of 0.5 and maximum iteration of 20.

Multiple imputation method

The pattern of missingness was arbitrary and variables with missing values were in continuous and categorical forms. Multiple imputation was thus performed by chained equation[3], which used a series of univariate conditional imputation models to impute missing values and Rubin's rule to combine the results[4]. The procedure was done under a missing at random (MAR) assumption. Continuous variables were imputed using linear regression models while categorical variables were imputed using logistic regression models. Based on the efficiency consideration that the variance of the multiple imputation estimate usually levels off as number of imputations (m) increases beyond 20, m ≤20 is deemed sufficient[4]. However, based on the rule of thumb that the number of imputations should at least equal the percentage of incomplete cases (which is 41.2% in our study), both 20 and 50 imputations were performed. As the results were similar, we used 20 imputations for our analysis.

Syntax for main analyses

Descriptive analysis and characteristics comparison:

```
FREQUENCIES VARIABLES=BAZ_wt_status_3gp_M12
  /ORDER=ANALYSIS.

ONEWAY Maternal_age_at_delivery z_score_xy_sex BAZ_M12 BY delivery_mode_3gp
  /STATISTICS DESCRIPTIVES
  /MISSING ANALYSIS
  /POSTHOC=TUKEY ALPHA(0.05).

CROSSTABS
  /TABLES=maternal_education_level ethnicity active_passive_during_pregnancy
    hypertensive_disorders_of_pregnancy GDM1999 child_gender child_feeding_first6months
    earlypreg_BMI_4cat_NL parity_2gp antibiotics_labour BY delivery_mode_3gp
  /FORMAT=AVALUE TABLES
  /STATISTICS=CHISQ
  /CELLS=COUNT COLUMN
  /COUNT ROUND CELL.

CROSSTABS
  /TABLES=BAZ_wt_status_3gp_M12 BY delivery_mode_3gp
  /FORMAT=AVALUE TABLES
  /STATISTICS=CHISQ
  /CELLS=COUNT COLUMN
  /COUNT ROUND CELL
  /METHOD=EXACT TIMER(5).
```

Logistic regression for model 2*:

```
LOGISTIC REGRESSION VARIABLES BAZ_ow_M12
  /METHOD=ENTER delivery_mode_3gp Maternal_age_at_delivery ethnicity
    maternal_education_level earlypreg_BMI_4cat_NL parity_2gp active_passive_during_pregnancy
    hypertensive_disorders_of_pregnancy GDM1999 z_score_xy_sex
```

© 2018 Cai M et al. *JAMA Network Open.*

```
 /CONTRAST (ethnicity)=Indicator(1)
 /CONTRAST (maternal_education_level)=Indicator(1)
 /CONTRAST (delivery_mode_3gp)=Indicator(1)
 /CONTRAST (earlypreg_BMI_4cat_NL)=Indicator(1)
 /CONTRAST (parity_2gp)=Indicator(1)
 /CONTRAST (active_passive_during_pregnancy)=Indicator(1)
 /CONTRAST (hypertensive_disorders_of_pregnancy)=Indicator(1)
 /CONTRAST (GDM1999)=Indicator(1)
 /PRINT=CI(95)
 /CRITERIA=PIN(0.05) POUT(0.10) ITERATE(20) CUT(0.5).
```

*Model 1 was ran with the same syntax minus the covariates, while model 3 included additional covariates "child_feeding_first6months" and "antibiotics_labour".

Multiple imputation:

```
Set SEED 29390.
DATASET DECLARE imputed.
MULTIPLE IMPUTATION BAZ_ow_M12 delivery_mode_3gp Maternal_age_at_delivery ethnicity
    maternal_education_level earlypreg_BMI_4cat_NL parity_2gp active_passive_during_pregnancy
    antibiotics_labour hypertensive_disorders_of_pregnancy GDM1999 z_score_xy_sex child_gender
    child_feeding_first6months
/IMPUTE METHOD=AUTO NIMPUTATIONS=20 MAXPCTMISSING=NONE
 /CONSTRAINTS BAZ_ow_M12 ( ROLE=IND)
 /CONSTRAINTS delivery_mode_3gp ( ROLE=IND)
 /MISSINGSUMMARIES NONE
 /IMPUTATIONSUMMARIES MODELS
 /OUTFILE IMPUTATIONS=imputed .
```

© 2018 Cai M et al. *JAMA Network Open.*

eTable 1. Comparison of maternal and infant characteristics among three subsets of participants.

Variables	No anthropometry data at age 12 months and/or lost to follow-up (n = 281)	Missing data on potential confounders and mediating factors (n = 229)	Included in primary analysis (n = 727)	*P*[a]
**Maternal**				
Ethnicity				.006
Chinese	137 (48.8)	143 (62.4)	411 (56.5)	
Malay	93 (33.1)	43 (18.8)	186 (25.6)	
Indian	51 (8.8)	43 (18.8)	130 (17.9)	
Missing	0 (0)	0 (0)	0 (0)	
Age at delivery, mean (SD), years	30.05 (5.28)	31.88 (4.71)	31.36 (5.15)	<.001
Missing	66	0	0	
Education				<.001
No-formal/Primary/Secondary	111 (39.5)	54 (23.6)	216 (29.7)	
Post-secondary	95 (33.8)	76 (33.2)	259 (35.6)	
University	68 (24.2)	89 (38.9)	252 (34.7)	
Missing	7 (2.5)	10 (4.4)	0 (0)	
Parity				.001
0	108 (38.4)	126 (55.0)	303 (41.7)	
≥ 1	108 (38.4)	103 (45.0)	424 (58.3)	
Missing	65 (23.1)	0 (0)	0 (0)	
Early pregnancy body mass index (BMI) status				.79
Underweight (<18.5 kg/m^2)	17 (6.0)	15 (6.6)	60 (8.3)	
Increasing but acceptable risk (18.5 ≤kg/m^2 <23)	88 (31.3)	73 (31.9)	339 (46.6)	
Increased risk (23 ≤kg/m^2 <27.5)	56 (19.9)	43 (18.8)	198 (27.2)	
High risk (≥27.5 kg/m^2)	27 (9.6)	40 (17.5)	130 (17.9)	
Missing	93 (33.1)	58 (25.3)	0 (0)	
Active or passive smoking during pregnancy				.01
No	104 (37.0)	130 (56.8)	407 (56.0)	
Yes	120 (42.7)	88 (38.4)	320 (44.0)	
Missing	57 (20.3)	11 (4.8)	0 (0)	
Hypertensive disorders of pregnancy				.50
No	259 (92.2)	210 (91.7)	681 (93.7)	
Yes	22 (7.8)	19 (8.3)	46 (6.3)	
Missing	0 (0)	0 (0)	0 (0)	
Gestational diabetes mellitus				.04
No	163 (58.0)	122 (53.3)	602 (82.8)	
Yes	37 (13.2)	42 (18.3)	125 (17.2)	
Missing	81 (28.8)	65 (28.4)	0 (0)	

[a]Chi-Square test for categorical variables and one-factor analysis of variance (ANOVA) for continuous variables were used to compare the 3 groups.

© 2018 Cai M et al. *JAMA Network Open.*

eTable 1. Comparison of maternal and infant characteristics among three subsets of participants (continued).

Variables	No anthropometry data at age 12 months and/or lost to follow-up (n = 281)	Missing data on potential confounders and mediating factors (n = 229)	Included in primary analysis (n = 727)	*P*[a]
Intrapartum antibiotics				.99
No	142 (50.5)	152 (66.4)	484 (66.6)	
Yes	73 (26.0)	76 (33.2)	243 (33.4)	
Missing	66 (23.5)	1 (0.4)	0 (0)	
Delivery mode				.62
Vaginal delivery	160 (56.9)	156 (68.1)	505 (69.5)	
Emergency caesarean delivery	38 (13.5)	50 (21.8)	148 (20.4)	
Elective caesarean delivery	17 (6.0)	23 (10.0)	74 (10.2)	
Missing	66 (23.5)	0 (0)	0 (0)	
Reclassified delivery mode				.61
Vaginal delivery	160 (56.9)	156 (68.1)	505 (69.5)	
Intrapartum caesarean delivery	18 (6.4)	24 (10.5)	78 (10.7)	
Non-labour caesarean delivery	37 (13.2)	49 (21.4)	144 (19.8)	
Missing	66 (23.5)	0 (0)	0 (0)	
**Infant**				
Sex				.30
Male	122 (43.4)	125 (54.6)	372 (51.2)	
Female	93 (33.1)	104 (45.4)	355 (48.8)	
Missing	66 (23.5)	0 (0)	0 (0)	
Sex-adjusted birth weight-for-gestational age z-score, mean (SD)	-0.11 (1.13)	0.31 (1.27)	0.17 (1.20)	.001
Missing	59	5	0	
Feeding during first 6 months				.19
Exclusive breastfeeding	8 (2.8)	21 (9.2)	96 (13.2)	
Mixed feeding	62 (22.1)	61 (26.6)	455 (62.6)	
Exclusive formula feeding	22 (7,8)	32 (14.0)	176 (24.2)	
Missing	189 (67.3)	115 (50.2)	0 (0)	
BMI-for-age z-score (BAZ) at age 12 months, mean (SD)	-1.65 (N.A.)	-0.18 (0.98)	-0.16 (1.03)	.34
Missing	280	0	0	

[a]Chi-Square test for categorical variables and one-factor analysis of variance (ANOVA) for continuous variables were used to compare the 3 groups.

© 2018 Cai M et al. *JAMA Network Open.*

eTable 2. Row percentages of categorical variables compared among different modes of delivery.

Variables[a]	Vaginal delivery (n = 505)	Emergency caesarean delivery (n = 148)	Elective caesarean delivery (n = 74)	*P*[b]
**Maternal**				
Ethnicity				.63
Chinese (n = 411)	293 (71.3)	76 (18.5)	42 (10.2)	
Malay (n = 186)	127 (68.3)	42 (22.6)	17 (9.1)	
Indian (n = 130)	85 (65.4)	30 (23.1)	15 (11.5)	
Maternal education				.62
No-formal/Primary/Secondary (n = 216)	151 (69.9)	44 (20.4)	21 (9.7)	
Post-secondary (n = 259)	173 (66.8)	60 (23.2)	26 (10.0)	
University (n = 252)	181 (71.8)	44 (17.5)	27 (10.7)	
Parity				<.001
0 (n = 303)	199 (65.7)	91 (30.0)	13 (4.3)	
≥ 1 (n = 424)	306 (72.2)	57 (13.4)	61 (14.4)	
Early pregnancy body mass index (BMI) status				.14
Underweight (<18.5 kg/m^2) (n = 60)	48 (80.0)	7 (11.7)	5 (8.3)	
Increasing but acceptable risk (18.5 ≤kg/m^2 <23) (n = 339)	239 (70.5)	72 (21.2)	28 (8.3)	
Increased risk (23 ≤kg/m^2 <27.5) (n = 198)	138 (69.7)	38 (19.2)	22 (11.1)	
High risk (≥27.5 kg/m^2) (n = 130)	80 (61.5)	31 (23.8)	19 (14.6)	
Active or passive smoking during pregnancy				.18
No (n = 407)	290 (71.3)	73 (17.9)	44 (10.8)	
Yes (n = 320)	215 (67.2)	75 (23.4)	30 (9.4)	
Hypertensive disorders of pregnancy				.006
No (n = 681)	477 (70.0)	131 (19.2)	73 (10.7)	
Yes (n = 46)	28 (60.9)	17 (37.0)	1 (2.2)	
Gestational diabetes mellitus				.57
No (n = 602)	423 (70.3)	120 (19.9)	59 (9.8)	
Yes (n = 125)	82 (65.6)	28 (22.4)	15 (12.0)	
Intrapartum antibiotics				<.001
No (n = 484)	342 (70.7)	76 (15.7)	66 (13.6)	
Yes (n = 243)	163 (67.1)	72 (29.6)	8 (3.3)	
**Infant**				
Sex				.31
Male (n = 372)	260 (69.9)	80 (21.5)	32 (8.6)	
Female (n = 355)	245 (69.0)	68 (19.2)	42 (11.8)	

[a]Values are shown as no. (row %).
[b]Chi-Square or Fisher's Exact tests for categorical variables were used to compare the 3 groups.

© 2018 Cai M et al. *JAMA Network Open.*

eTable 2. Row percentages of categorical variables compared among different modes of delivery (continued).

Variables[a]	Vaginal delivery (n = 505)	Emergency caesarean delivery (n = 148)	Elective caesarean delivery (n = 74)	*P*[b]
Feeding during first 6 months				.16
Exclusive breastfeeding (n = 96)	73 (76.0)	12 (12.5)	11 (11.5)	
Mixed feeding (n = 455)	318 (69.9)	91 (20.0)	46 (10.1)	
Exclusive formula feeding (n = 176)	114 (64.8)	45 (25.6)	17 (9.7)	
BMI status at age 12 months				.12
Not at risk of overweight (BAZ ≤1 SD) (n = 629)	439 (70.7)	126 (20.3)	56 (9.0)	
At risk of overweight (1 SD <BAZ ≤2 SDs) (n = 81)	55 (61.8)	18 (20.2)	16 (18.0)	
Overweight (BAZ >2 SDs) (n = 17)	11 (64.7)	4 (23.5)	2 (11.8)	

[a]Values are shown as no. (row %).
[b]Chi-Square or Fisher's Exact tests for categorical variables were used to compare the 3 groups.

© 2018 Cai M et al. *JAMA Network Open.*

eTable 3. Association of intrapartum and non-labour caesarean delivery with risk of overweight/overweight at age 12 months[a] (n = 727).

	Model 1[b]		Model 2		Model 3	
	OR (95% CI)	*P*	OR (95% CI)	*P*	OR (95% CI)	*P*
**Delivery mode**						
Vaginal delivery	1.0	-	1.0	-	1.0	-
Intrapartum caesarean delivery	0.87 (0.41, 1.82)	.71	0.66 (0.30, 1.47)	.31	0.68 (0.30, 1.55)	.36
Non-labour caesarean delivery	1.83 (1.14, 2.93)	.01	1.63 (0.98, 2.71)	.06	1.63 (0.98, 2.72)	.06

[a]Data were analysed using logistic regression.
[b]Model 1: Crude (unadjusted).
Model 2: Adjusted for ethnicity, maternal age at delivery, maternal educational level, parity, early pregnancy BMI, antenatal active/passive smoking, hypertensive disorders of pregnancy, gestational diabetes, and sex-adjusted birth weight-for-gestational age z-score.
Model 3: Model 2 + Adjusted for intrapartum antibiotics and infant feeding during first 6 months.

© 2018 Cai M et al. *JAMA Network Open.*

eTable 4. Association of delivery mode with continuous outcome variable BMI z-score (BAZ) at age 12 months[a] (n = 727).

	Model 1[b]		Model 2		Model 3	
	β (95% CI)	*P*	β (95% CI)	*P*	β (95% CI)	*P*
**Delivery mode**						
Vaginal delivery	Reference	-	Reference	-	Reference	-
Emergency caesarean delivery	0.00 (-0.19, 0.19)	.99	-0.03 (-0.27, 0.10)	.37	-0.03 (-0.26, 0.11)	.43
Elective caesarean delivery	0.09 (0.07, 0.57)	.01	0.08 (0.02, 0.50)	.04	0.08 (0.02, 0.51)	.04

[a]Data were analysed using linear regression.
[b]Model 1: Crude (unadjusted).
Model 2: Adjusted for ethnicity, maternal age at delivery, maternal educational level, parity, early pregnancy BMI, antenatal active/passive smoking, hypertensive disorders of pregnancy, gestational diabetes, and sex-adjusted birth weight-for-gestational age z-score.
Model 3: Model 2 + Adjusted for intrapartum antibiotics and infant feeding during first 6 months.

© 2018 Cai M et al. *JAMA Network Open.*

eTable 5. Association of delivery mode with BMI status at age 12 months in ordinal form[a] (n = 727).

	Model 1[b]		Model 2		Model 3	
	OR (95% CI)	*P*	OR (95% CI)	*P*	OR (95% CI)	*P*
**Delivery mode**						
Vaginal delivery	1.0	-	1.0	-	1.0	-
Emergency caesarean delivery	1.09 (0.82, 1.43)	.56	0.98 (0.73, 1.32)	.89	0.99 (0.73, 1.33)	.92
Elective caesarean delivery	1.46 (1.05, 2.05)	.03	1.46 (1.03, 2.09)	.04	1.47 (1.03, 2.11)	.04

[a]BMI status was analysed in ordinal form (i.e. not at risk of overweight, at risk overweight, overweight) using ordinal logistic regression (Probit link).
[b]Model 1: Crude (unadjusted).
Model 2: Adjusted for ethnicity, maternal age at delivery, maternal educational level, parity, early pregnancy BMI, antenatal active/passive smoking, hypertensive disorders of pregnancy, gestational diabetes, and sex-adjusted birth weight-for-gestational age z-score.
Model 3: Model 2 + Adjusted for intrapartum antibiotics and infant feeding during first 6 months.

© 2018 Cai M et al. *JAMA Network Open.*

eTable 6. Association of delivery mode with risk of overweight/overweight at age 12 months stratified according to parity.

**Nulliparous (n = 303)**						
	Model 1[b]		Model 2		Model 3	
	OR (95% CI)	*P*	OR (95% CI)	*P*	OR (95% CI)	*P*
**Delivery mode**						
Vaginal delivery	1.0	-	1.0	-	1.0	-
Emergency caesarean delivery	0.47 (0.21, 1.06)	.07	0.27 (0.11, 0.68)	.005	0.25 (0.10, 0.65)	.005
Elective caesarean delivery	0.40 (0.05, 3.22)	.39	0.26 (0.02, 3.24)	.30	0.31 (0.02, 3.89)	.36
**Parity ≥1 (n = 424)**						
	Model 1[b]		Model 2		Model 3	
	OR (95% CI)	*P*	OR (95% CI)	*P*	OR (95% CI)	*P*
**Delivery mode**						
Vaginal delivery	1.0	-	1.0	-	1.0	-
Emergency caesarean delivery	2.79 (1.38, 5.65)	.004	2.62 (1.22, 5.60)	.01	2.91 (1.34, 6.32)	.007
Elective caesarean delivery	3.31 (1.70, 6.46)	<.001	3.45 (1.68, 7.07)	.001	3.21 (1.55, 6.68)	.002

[a]Data were analysed using logistic regression.
[b]Model 1: Crude (unadjusted).
Model 2: Adjusted for ethnicity, maternal age at delivery, maternal educational level, early pregnancy BMI, antenatal active/passive smoking, hypertensive disorders of pregnancy, gestational diabetes, and sex-adjusted birth weight-for-gestational age z-score.
Model 3: Model 2 + Adjusted for intrapartum antibiotics and infant feeding during first 6 months.

© 2018 Cai M et al. *JAMA Network Open.*

eReferences

1. World Health Organization Expert Consultation. Appropriate body-mass index for Asian populations and its implications for policy and intervention strategies. *Lancet.* 2004;363(9403):157-163.
2. Alberti KG, Zimmet PZ. Definition, diagnosis and classification of diabetes mellitus and its complications. Part 1: diagnosis and classification of diabetes mellitus provisional report of a WHO consultation. *Diabetic medicine : a journal of the British Diabetic Association.* 1998;15(7):539-553.
3. Royston P. Multiple imputation of missing values. The Stata Journal. 2004;4(3):227-241.
4. Rubin DB. Multiple imputation for nonresponse in surveys. In: John Wiley & Sons, Inc; 2008: http://onlinelibrary.wiley.com/book/10.1002/9780470316696.

© 2018 Cai M et al. *JAMA Network Open.*
